# A rare case of dual congenital coronary cameral fistula and myocardial bridge: A case report

**DOI:** 10.1097/MD.0000000000028952

**Published:** 2022-04-22

**Authors:** Yong Shen

**Affiliations:** Department of Internal Medicine-Cardiovascular, Hechi People's Hospital, Hechi, Guangxi, China.

**Keywords:** β-blockers, conservative treatment, coronary artery fistula, myocardial bridge

## Abstract

**Rationale:**

A coronary artery fistula (CAF) is an anomalous communication between a coronary artery and a cardiac chamber or great vessel. It is a rare congenital anomaly that is often small and asymptomatic, occurring in only 0.002% of the general population. Most CAFs originate from the right coronary artery and flow into the right cardiac system. Although extremely rare, some cases may originate from the bilateral coronary arteries and flow into the left ventricle.

**Patient concerns:**

Herein, we report a rare case of a 55-year-old male smoker with no history of heart disease or cardiac surgery, who presented with a 5-year history of recurrent chest congestion, palpitations, and shortness of breath. On physical examination, his heart and lungs revealed normal findings without cardiac murmurs and no systemic or pulmonary edema. Moreover, 24-hour ambulatory electrocardiography showed no signs of ischemia but exhibited a short array of ventricular tachycardia and short atrial tachycardia. Chest computed tomography showed left apical emphysema without cardiomegaly and pulmonary congestion. Furthermore, coronary angiography revealed dual congenital coronary cameral fistula, a complex CAF with a left circumflex artery–left ventricle fistula and a right coronary artery–left ventricle fistula, complicated with a myocardial bridge.

**Diagnosis and interventions:**

A diagnosis of left circumflex artery–left ventricle fistula complicated with a right coronary artery–left ventricle fistula and myocardial bridge was made. Since the patient refused surgery, medical management with enteric-coated aspirin, sustained-release metoprolol, and atorvastatin calcium was initiated.

**Outcomes and lesson:**

Currently, the patient is now asymptomatic and in good condition since 6 months after undergoing conservative treatment with β-blockers.

## 1 Introduction

A coronary artery fistula (CAF) is an abnormal connection between a coronary artery and a cardiac chamber or great vessel,^[1]^ as first described by Krause in 1865.^[2]^ It can be congenital or acquired and has an estimated prevalence of 0.002% in the general population and 0.25% to 0.5% in patients who undergo coronary angiography.^[3]^ Most CAFs originate from the right coronary artery and flow into the right cardiac system.^[1]^ Although extremely rare, CAFs may originate from the bilateral coronary arteries and flow into the left ventricle.^[4]^

Herein, we report a rare case of dual congenital coronary cameral fistula, a complex CAF with a left circumflex artery–left ventricle fistula and a right coronary artery–left ventricle fistula, complicated with a myocardial bridge. This case can provide some lessons for clinicians and increase awareness of these conditions and associated diseases.

## 2 Case presentation

**Figure 1:**
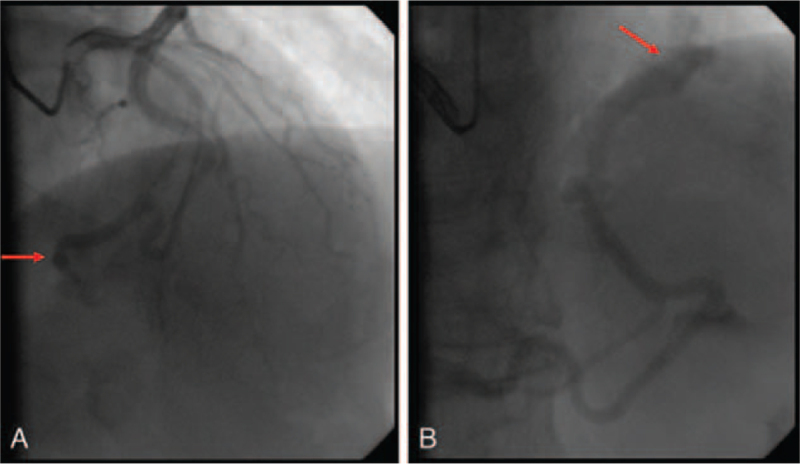
Coronary angiography showing a left circumflex artery–left ventricle fistula (A) complicated with a right coronary artery–left ventricle fistula (B).

Our patient was a 55-year-old male smoker with no history of heart disease or cardiac surgery, who presented with a 5-year history of recurrent palpitations, chest congestion, and shortness of breath. Notably, there was no apparent correlation between the palpitations and his daily activities. On admission, his blood pressure and heart rate were 121/70 mm Hg and 73 beats/min, respectively. Physical examination of his heart and lungs revealed normal findings without cardiac murmurs and systemic or pulmonary edema. Furthermore, his levels of troponin, myoglobin, creatine kinase, creatine kinase-MB, type B natriuretic peptide, and serum creatinine were all normal. To identify if his symptoms were caused by respiratory or cardiovascular diseases, we performed 24-hour ambulatory electrocardiography, coronary angiography, and chest computed tomography. On analysis, 24-hour ambulatory electrocardiography showed no signs of ischemia but exhibited a short array of ventricular tachycardia and short atrial tachycardia. Chest computed tomography also showed left apical emphysema without cardiomegaly and pulmonary congestion. In addition, cardiac color ultrasonography revealed ventricular diastolic compliance that impedes diastolic dysfunction and with an ejection fraction of 68%, as well as mild aortic and tricuspid regurgitation. Accordingly, coronary angiography was performed, revealing a CAF originating from the left anterior descending artery draining into the left ventricle (Fig. 1A, Video S1, Supplemental Digital Content) with a right coronary artery–left ventricle fistula (Fig. 1B, Video S2, Supplemental Digital Content) without significant coronary obstruction. However, a myocardial bridge in the middle of the left anterior descending artery compressing approximately 70% of the left anterior descending artery during cardiac contraction was also observed (Video S1, Supplemental Digital Content). Therefore, a diagnosis of left circumflex artery–left ventricle fistula and a right coronary artery–left ventricle fistula complicated with a myocardial bridge was made. Since the patient had bilateral coronary artery–left ventricular fistulas with 2 drainage points, CAF ligation under thoracotomy was recommended. However, the patient refused the surgery due to economic reasons and opted for medical management with enteric-coated aspirin, sustained-release metoprolol, and atorvastatin calcium. The patient's palpitations was then resolved with significant improvement before being discharged, and he continued oral medications at home. At the 6-month follow-up, the patient remained asymptomatic without findings of angina pectoris, myocardial infarction, or heart failure. Written informed consent was obtained from the patient for the publication of this report.

## 3 Discussion

Congenital heart disease is the primary etiology for 90% of CAFs, which is possibly caused by infections during early embryonic development or genetic factors.^[5]^ Recently, with the increase in traumatic events and improvements in invasive procedures, including myocardial biopsy, coronary stent implantation, coronary artery bypass surgery, chest irradiation, and thoracotomy, the incidence of acquired CAF has increased as well.^[2]^

CAF can originate from any side of the coronary artery, with the most common site being a single CAF. More specifically, the right coronary artery is the most common origin (50%–60%), followed by the left coronary artery (35%–42%). CAFs originating from both sides are possible but rare, accounting for approximately 5% of CAFs. Moreover, over 90% of CAFs terminate into the right heart (approximately 41% in the right ventricle, 26% in the right atrium), whereas only 3% terminate into the left heart.^[4,6]^ In this case, the patient did not have a history of cardiovascular surgery or cardiac catheterization. Thus, CAF due to congenital malformations was likely. Furthermore, the patient's CAF originated from bilateral coronary arteries (left circumflex artery and right coronary artery) and both terminated into the left ventricle, which is extremely rare.

The clinical presentation, symptomatology, and influence of CAF on hemodynamics depend on its size, shunt direction, and location.^[7]^ Most patients with CAFs remain asymptomatic in the first 2 decades of life, especially in patients with smaller shunt volumes that are incidentally found. However, as the size of the fistula increases, more hemodynamic effects occur. Progressive enlargement of a fistula may result in a prime pathologically significant large fistula, called “coronary steal,” causing myocardial hypoperfusion distal to the fistulous connection.^[7]^ In severe cases, pulmonary hypertension, ruptured aneurysm, heart failure, arrhythmia, myocardial infarction, newly infected endocarditis, sudden cardiac death, and other severe complications may occur. However, in typical cases, patients experience exertional dyspnea, fatigue, angina, or palpitations.^[8]^

Spontaneous closure of CAF may occur in approximately 1% of patients. For most cases, CAF management includes conservative treatment, medical therapy, transcatheter intervention, and surgery.^[4,8]^ Regarding surgery, selection of the CAF closure technique depends on morphology, course, tortuosity, and the presence of an aneurysmal dilatation in the vessel that supplies blood and the vessel earmarked for surgical closure. However, for clinically silent and hemodynamically insignificant fistulas, treatment is usually not required. The basic principle for all surgical treatments is to block the fistula and restore normal coronary circulation before patients develop severe complications.^[3]^ The American College of Cardiology/American Heart Association^[1]^ guidelines for the management of adults with congenital heart diseases recommend cardiac catheterization or surgical intervention for all large fistulas, regardless of their symptomatology. For small to medium-sized fistula with obvious symptoms, intervention is also indicated.^[9]^ However, there is no consensus on the management of asymptomatic aneurysms or any clinical consensus on the indications for conservative treatment. Antiplatelet therapy is usually recommended for conservative treatment to prevent local fistula-induced vortices from increasing thrombus formation, and β-blockers and calcium channel blockers are also used accordingly. Regardless of which management is used, long-term follow-up is vital to assess the prognosis of patients after treatment.

In our case, we discovered a CAF originating from the left anterior descending artery and draining into the left ventricle, which was complicated with a right coronary artery–left ventricle fistula. Left-to-left shunts and right-to-left shunts can form pathophysiological changes similar to those of aortic insufficiency. In cases of large shunt volumes, there may be increased volume load and left ventricular enlargement, thereby resulting in congestive heart failure. In addition, there was a myocardial bridge in the middle of the left anterior descending artery, compressing approximately 70% of it during cardiac contraction. Thus, the coronary blood supply in this patient was impaired during cardiac contraction and diastole. Due to economic reasons, the patient refused cardiac catheterization or surgical intervention and opted for medical therapy. Interestingly, the palpitations of the patients significantly improved and subsequently disappeared after conservative treatment. This may be related to the control of the heart rate, relaxation of the myocardium, and improvement of coronary microcirculation by β-receptor blockers. However, in recent years, propranolol hydrochloride, a non-selective blocker, has been observed to produce dramatic involution of infantile hemangiomas.^[10]^

## Author contributions

**Conceptualization:** Yong Shen.

**Data curation:** Yong Shen.

**Investigation:** Yong Shen.

**Writing – original draft:** Yong Shen.

**Writing – review & editing:** Yong Shen.

## Supplementary Material

Supplemental Digital Content

## Supplementary Material

Supplemental Digital Content
